# Narcolepsy and rapid eye movement sleep

**DOI:** 10.1111/jsr.14277

**Published:** 2024-07-02

**Authors:** Francesco Biscarini, Lucie Barateau, Fabio Pizza, Giuseppe Plazzi, Yves Dauvilliers

**Affiliations:** ^1^ Department of Biomedical and Neuromotor Sciences (DIBINEM) University of Bologna Bologna Italy; ^2^ IRCCS Istituto delle Scienze Neurologiche di Bologna Bologna Italy; ^3^ Sleep‐Wake Disorders Unit, Department of Neurology Gui‐de‐Chauliac Hospital, CHU Montpellier Montpellier France; ^4^ National Reference Centre for Orphan Diseases, Narcolepsy, Idiopathic Hypersomnia, and Kleine‐Levin Syndrome Montpellier France; ^5^ Institute for Neurosciences of Montpellier University of Montpellier, INSERM Montpellier France; ^6^ Department of Biomedical, Metabolic and Neural Sciences University of Modena and Reggio‐Emilia Modena Italy

**Keywords:** cataplexy, history, hypersomnia, orexin/hypocretin, sleep‐onset rapid eye movement period

## Abstract

Since the first description of narcolepsy at the end of the 19th Century, great progress has been made. The disease is nowadays distinguished as narcolepsy type 1 and type 2. In the 1960s, the discovery of rapid eye movement sleep at sleep onset led to improved understanding of core sleep‐related disease symptoms of the disease (excessive daytime sleepiness with early occurrence of rapid eye movement sleep, sleep‐related hallucinations, sleep paralysis, rapid eye movement parasomnia), as possible dysregulation of rapid eye movement sleep, and cataplexy resembling an intrusion of rapid eye movement atonia during wake. The relevance of non‐sleep‐related symptoms, such as obesity, precocious puberty, psychiatric and cardiovascular morbidities, has subsequently been recognized. The diagnostic tools have been improved, but sleep‐onset rapid eye movement periods on polysomnography and Multiple Sleep Latency Test remain key criteria. The pathogenic mechanisms of narcolepsy type 1 have been partly elucidated after the discovery of strong HLA class II association and orexin/hypocretin deficiency, a neurotransmitter that is involved in altered rapid eye movement sleep regulation. Conversely, the causes of narcolepsy type 2, where cataplexy and orexin deficiency are absent, remain unknown. Symptomatic medications to treat patients with narcolepsy have been developed, and management has been codified with guidelines, until the recent promising orexin‐receptor agonists. The present review retraces the steps of the research on narcolepsy that linked the features of the disease with rapid eye movement sleep abnormality, and those that do not appear associated with rapid eye movement sleep.

## 1 INTRODUCTION

Narcolepsy is a rare neurological disorder nowadays classified into two distinct forms, narcolepsy type 1 (NT1) and type 2 (NT2; Barateau et al., 2023). The prevalence ranges between 0.026 and 0.052/100,000 people across different ethnic groups (Silber et al., 2002), and narcolepsy is probably underdiagnosed. The typical age of onset is bimodal, with the major peak of incidence from childhood to early adulthood, and the second peak in the third decade (Bassetti et al., 2019; Dauvilliers et al., 2001).

Narcolepsy was first described between 1877 and 1880 in the works of Westphal, Fisher and Gélineau, as a disease characterized by excessive daytime sleepiness (EDS) with irresistible sleep attacks and by brief episodes of loss of muscle tone triggered by emotions without altered consciousness, named cataplexy (Fischer, 1878; Gelineau, 1880; Westphal, 1877).

In 1960, 7 years after Aserinsky and Kleitman recorded rapid eye movement (REM) sleep (Aserinsky & Kleitman, 1953), Gerald Vogel, while studying dreams in patients with narcolepsy, described precocious transitions from wakefulness to REM sleep at the onset of nocturnal sleep (Vogel, 1960). This abnormal early appearance of REM sleep, defined as sleep‐onset REM period (SOREMP), was confirmed by Rechtschaffen et al. in 1963 and Passouant et al. in 1964 (Passouant et al., 1964; Rechtschaffen et al., 1963), and became the key neurophysiological marker of narcolepsy. SOREMPs were then recorded during the daytime sleep of patients with narcolepsy (Wilson et al., 1973) and during the Multiple Sleep Latency Test (MSLT; Mitler et al., 1979).

Several core clinical features of narcolepsy, such as cataplexy, sleep paralysis and hypnagogic/hypnopompic hallucinations, are thought to be manifestations of dissociation of behavioural states, with elements of REM sleep intruding wakefulness (Bassetti et al., 2019).

Dogs with familial narcolepsy, with cataplexy and SOREMPs, have been described since 1973 (Knecht et al., 1973; Mitler et al., 1974), allowing, in 1999, the identification of mutations of the orexin/hypocretin receptor‐2 gene as the cause of canine narcolepsy (Lin et al., 1999). In contrast, in humans, the strong association between polymorphisms of HLA class II and narcolepsy, widely described in different populations since 1984, suggested an immune‐mediated cause of the disease (Billiard et al., 1986; Juji et al., 1984; Langdon et al., 1984).

In 1990, the first edition of the International Classification of Sleep Disorders (ICSD) included cataplexy and other REM sleep phenomena as criteria for narcolepsy, together with the presence of SOREMPs (Diagnostic Classification Steering Committee, 1990). In 2000, the acquired loss of orexin was demonstrated to cause narcolepsy with cataplexy in humans. In the second edition of ICSD in 2005 (ICSD‐2), narcolepsy was divided into narcolepsy with cataplexy and narcolepsy without cataplexy, both distinguished from idiopathic hypersomnia (IH), and orexin deficiency was accounted as a confirmatory criterion of narcolepsy with cataplexy, when a lumbar puncture is performed (American Academy of Sleep Medicine, 2005). The 2014 third edition of ICSD (ICSD‐3) re‐defined the entities of NT1 and NT2. NT1 is defined by sleepiness with either: (i) cataplexy and MSLT with mean sleep latency ≤ 8 min and at least two SOREMPs (counting also the possible SOREMP during the night preceding MSLT); or (ii) cerebrospinal fluid (CSF) orexin deficiency < 110 pg ml^−1^. Conversely, NT2 is defined by chronic sleepiness confirmed by pathological MSLT (with the same values of NT1) not better explained by other causes, without cataplexy and without orexin deficiency (if measured; American Academy of Sleep Medicine, 2014). In 2023, the ICSD‐3 text revision (ICSD‐3‐TR) defined the presence of the night‐SOREMP combined with cataplexy sufficient for diagnosing NT1, regardless of MSLT, while NT2 criteria remained unchanged (American Academy of Sleep Medicine, 2023). Different sets of ICSD criteria for narcolepsy are reported in Table 1. Even though the disease goes beyond a “simple” dysregulation of REM sleep, the role of REM sleep and how the orexin deficiency impairs its physiological mechanisms remains of key interest in the research on narcolepsy. Moreover, the mechanisms of the REM sleep dysfunction observed in NT2, without orexin deficiency, remain unclear and to be explored.

**TABLE 1:** Evolution of definitions of narcolepsy in the ICSD editions

ICSD, 1990 (diagnostic classification steering Committee, 1990)	
Narcolepsy	
A complaint of excessive sleepiness or sudden muscle weakness Recurrent daytime naps or lapses into sleep that occur almost daily for at least 3 months Sudden bilateral loss of postural muscle tone in association with intense emotion (cataplexy) Associated features include: Sleep paralysis Hypnagogic hallucinations Automatic behaviours Disrupted major sleep episode PSG demonstrates one or more of the following: Sleep latency <10 min REM sleep latency < 20 min; and MSLT that demonstrates a mean sleep latency of < 5 min Two or more SOREMPs HLA typing demonstrates DR2 positivity Absence of any medical or psychiatric disorder that could account for the symptoms Other sleep disorders may be present, but are not the primary cause of the symptoms	

ICSD‐2 (American Academy of Sleep Medicine, 2005)	
Narcolepsy with Cataplexy	Narcolepsy without Cataplexy
A–D must be met The patient has a complaint of EDS occurring almost daily for at least 3 months A definite history of cataplexy, defined as sudden and transient episodes of loss of motor tone triggered by emotions is present The diagnosis of narcolepsy with cataplexy should, whenever possible, be confirmed by nocturnal PSG followed by MSLT; the mean sleep latency on MSLT is less than or equal to 8 min and two or more SOREMPs are observed following sufficient nocturnal sleep (minimum 6 hr) during the night prior to the test. Alternatively, hypocretin‐1 levels in the CSF are less than or equal to 110 pg ml^−1^ or one‐third of mean normal values The hypersomnia is not better explained by another sleep disorder or neurological disorder, mental disorder, medication use, or substance use disorder	A–D must be met The patient has a complaint of EDS occurring almost daily for at least 3 months Typical cataplexy is not present, although doubtful or atypical cataplexy‐like episodes may be reported The diagnosis of narcolepsy without cataplexy must be confirmed by nocturnal PSG followed by MSLT. In narcolepsy without cataplexy, the mean sleep latency on MSLT is less than or equal to 8 min and two or more SOREMPs are observed following sufficient nocturnal sleep (minimum 6 h) during the night prior to the test The hypersomnia is not better explained by another sleep disorder, medical or neurological disorder, mental disorder, medication use, or substance use disorder

ICSD‐3, 2014 (American Academy of Sleep Medicine, 2014)	
NT1	NT2
A–B must be met The patient has daily periods of irrepressible need to sleep or daytime lapses into sleep occurring for at least 3 months The presence of one or both of the following: Cataplexy (as defined under *Essential Features –* see Table 2) *and* a mean sleep latency of ≤ 8 min and two or more SOREMPs on an MSLT performed according to standard techniques. A SOREMP (within 15 min of sleep onset) on the preceding nocturnal PSG may replace one of the SOREMPs on the MSLT CSF hypocretin‐1 concentration, measured by immunoreactivity, is either ≤ 110 pg ml^−1^ or < 1/3 of mean values obtained in normal subjects with the same standardized assay	A–E must be met The patient has daily periods of irrepressible need to sleep or daytime lapses into sleep occurring for at least 3 months A mean sleep latency of ≤ 8 min and two or more SOREMPs are found on a MSLT performed according to standard techniques. A SOREMP (within 15 min of sleep onset) on the preceding nocturnal PSG may replace one of the SOREMPs on the MSLT Cataplexy is absent Either CSF hypocretin‐1 concentration has not been measured *or* CSF hypocretin‐1 concentration measured by immunoreactivity is either > 110 pg ml^−1^ *or* > 1/3 of mean values obtained in normal subjects with the same standardized assay The hypersomnolence and/or MSLT findings are not better explained by other causes such as insufficient sleep, OSA, delayed sleep phase disorder, or the effect of medication or substances or their withdrawal

ICSD‐3‐TR, 2023 (American Academy of Sleep Medicine, 2023)	
NT1	NT2
A–B must be met The patient has daily periods of irrepressible need to sleep or daytime lapses into drowsiness or sleep The presence of one or both of the following elements: Cataplexy (Table 2), and either: Mean sleep latency of ≤ 8 min and two or more SOREMPs on a MSLT performed in accordance with current recommended protocols; or SOREMP (within 15 min of sleep onset) on nocturnal PSG CSF orexin‐A/hypocretin‐1 level, measured by RIA, is ≤ 110 pg ml^−1^ (using a Stanford reference sample) or less than one‐third of mean values obtained in normal subjects with the same standardized immunoreactivity assay	A–E, unchanged from ICSD‐3

Abbreviations: CSF, cerebrospinal fluid; EDS, excessive daytime sleepiness; ICSD, International Classification of Sleep Disorders; ICSD‐3‐TR, 3rd edition of International Classification of Sleep Disorders text revision; MSLT, Multiple Sleep Latency Test; NT1, narcolepsy type 1; NT2, narcolepsy type 2; OSA, obstructive sleep apnea; PSG, polysomnography; REM, rapid eye movement; RIA, radioimmunoassay; SOREMP, sleep‐onset rapid eye movement period.

## 2 MAIN FEATURES OF NARCOLEPSY, RELATED AND UNRELATED TO REM SLEEP DYSREGULATION

Historically, narcolepsy was defined by a tetrad of symptoms consisting of EDS, cataplexy, hypnagogic and hypnopompic hallucinations, and sleep paralysis (Yoss & Daly, 1957). Nowadays, disturbed nocturnal sleep (DNS) is recognized as another core feature of the pentad of NT1 (Maski et al., 2022). Cataplexy is present only in NT1, by definition. The other core symptoms seem more frequent and more severe in NT1 than in NT2 (Barateau et al., 2024; Luca et al., 2013; Zhang et al., 2018). NT1 presents a subacute or chronic onset and a life‐long chronic course, though the severity of symptoms can change over the years. The clinical history of NT2 can be more variable: symptoms can persist or can be transient, and cataplexy can develop years after EDS onset (thus reclassifying the patient as NT1; Fronczek et al., 2020). Overall, narcolepsy symptoms may change over the lifespan, but few data are available to date (Lividini et al., 2021).

### 2.1 Sleepiness

The EDS often emerges as the first and most disabling symptom of the disease, with some peculiar characteristics. Particularly noteworthy in NT1, EDS is often described as irresistibly compelling, leading to sudden sleep occurrences, even during active tasks. These brief periods of sleep are typically restorative and may include oneiric activity, considered the hallmark of early transition to REM sleep (Dement et al., 1966). Dreams during daytime SOREMPs in narcolepsy present narrative characteristics comparable to nocturnal dreams (Cipolli et al., 2020). Naps in narcolepsy do not necessarily present SOREMPs (Luca et al., 2013), suggesting that dysfunction of REM sleep does not entirely explain falling asleep. Severe EDS can manifest through automatic behaviours during wakefulness, typical but not specific for narcolepsy, where individuals unconsciously continue activities without subsequent memory of the events (Barateau et al., 2023; Guilleminault et al., 1975).

### 2.2 Cataplexy

Cataplexy, the pathognomonic symptom of NT1, is characterized by sudden and transient, usually brief, loss of muscle tone, triggered by emotional stimuli (typically positive ones). It can be both generalized and partial, variable in the distribution, duration and frequency of episodes (Bassetti et al., 2019; Dauvilliers et al., 2014). In paediatric cases of NT1, cataplexy can appear as persistent fluctuating hypotonia defined status cataplecticus typically involving the facial district (facies cataplectica) in the absence of emotional stimulation associated with hyperkinetic phenomena superimposed on the atonia (Plazzi et al., 2011). Cataplectic attacks are rarely observed in clinical practice at in‐office consultations, therefore their diagnosis is based mostly on characteristics reported by the patient, to be interpreted according to degrees of certainty (Table 2; American Academy of Sleep Medicine, 2023). However, attacks may be easily elicited by showing funny videos (Vandi, Pizza et al., 2019), allowing the identification of facial characteristics useful to distinguish cataplexy from functional neurological disorders (Pizza et al., 2018). The cataplectic attack is considered an abnormal intrusion of muscle atonia typical of REM sleep into wakefulness (Bassetti et al., 2019; Dement et al., 1966). Video‐polygraphic recordings of episodes can show a transient decrease in muscle activity that, in generalized attacks, can be accompanied by electroencephalography (EEG) desynchronization with possible sawtooth waves, and by bursts of rapid eye movements, suggesting that multiple features of REM sleep besides atonia appear during the attack. In contrast with physiological REM sleep, which presents sympathetic activation with unstable blood pressure and heart rate, generalized cataplexy has been described with associated bradycardia with stable blood pressure (Rubboli et al., 2000; Vetrugno et al., 2010). The occurrence of a burst of theta activity during cataplexy has been reported in children, as in the animal model (Bastianini et al., 2012; Vassalli et al., 2013).

**TABLE 2:** Description of cataplexy in the ICSD editions.

ICSD‐3, 2014 (American Academy of Sleep Medicine, 2014)	
*Essential features*: Cataplexy is defined as more than one episode of generally brief (<2 min), usually bilaterally symmetrical sudden loss of muscle tone with retained consciousness. The episodes are precipitated by strong emotions, usually positive, with almost all patients reporting some episodes precipitated by emotions associated with laughter.	

ICSD‐3 Text Revision, 2023 (American Academy of Sleep Medicine, 2023)	
Clinical features *Typical cataplectic attacks* ‐ Type of attack: partial (most often) and generalized (sometimes) loss of muscle tone *OR* only partial loss of muscle tone ‐ Localization Partial: bilateral loss of muscle tone in the face, neck, or legs, sometimes arms Generalized: bilateral progressive loss of muscle tone (generally starts in the face or neck and builds over seconds, leading to inability to stand and sometimes a subsequent fall) ‐ Abrupt return of muscle tone after the attack ‐ Triggers: clear emotional triggers; sudden positive emotions as laughter, telling a joke, making a witty remark, surprise; less frequently anger ‐ Duration: brief, up to 1 min, usually <30 s, classically a few seconds ‐ Frequency: variable, but usually at least once per month if untreated ‐ Consciousness preserved ‐ Cataplectic rebound when an antidepressant agent is abruptly interrupted *Variation of typical cataplexy* Occasional spontaneous episodes (especially when the patient is sleepy, and quite common in children); asymmetrical attack; facial twitching; jerky arm movements; rare episodes, less than one per year, if untreated *Specificities in children* ‐ Cataplectic facies: subtle facial expressions or choreic‐like movements, perioral movements such as grimacing and tongue protrusion, droopy eyelids, mouth opening ‐ Generalized hypotonia (gait unsteadiness), or occurrence of falls in the absence of clear emotional triggers (i.e. spontaneous episodes) ‐ Negative and active motor disturbances, with complex hyperkinetic component ‐ Episodes not always related to emotions ‐ Lack of awareness, movements reported by parents only	Degrees of Certainty ‐ Highly probable: two or more of the characteristics of “typical cataplectic attacks” ‐ Probable: variations of typical cataplexy ‐ Possible: at least one of the characteristics of “atypical cataplectic attacks” ‐ Unlikely: one or more of the characteristics of “atypical cataplectic attacks” ‐ Very unlikely: at least one of the characteristics listed in “episodes not compatible with cataplexy”

### 2.3 Sleep‐related hallucinations and paralysis

Hypnagogic and hypnopompic hallucinations were found in about 80% of NT1 and 40% of NT2, and sleep paralysis in 59% of NT1 and 28% of NT2 (Barateau et al., 2024; Dauvilliers, Barateau et al., 2020). These symptoms mostly represent state dissociation between elements of REM sleep and wakefulness (Antelmi et al., 2016; Bassetti et al., 2019). Sleep paralysis consists of generalized muscle atonia combined with wakefulness, occurring during the transition from wake to REM sleep or REM sleep to wake (Dauvilliers, Billiard et al., 2003). REM sleep and wake EEG activities can coexist with paralysis (Terzaghi et al., 2012). Hypnagogic hallucinations – visual, auditory, tactile or multimodal – appear more often associated with SOREMPs and can be considered dream‐like mental content emerging at the wake–sleep transition (Dauvilliers, Billiard et al., 2003). Although usually mild and perceived as “not real”, they can be vivid and disturbing so that patients can interpret them in a delusion‐like way misdiagnosed as psychotic disorder (Hanin et al., 2021; Jardri et al., 2014). Hallucinations in narcolepsy are most often related to sleep onset/offset and are multisensorial, while in psychotic disorders are mainly unimodal auditory (Dahmen et al., 2002; Plazzi et al., 2015). Interestingly, some studies suggested that the presence of hypnagogic hallucinations and other REM sleep‐related symptoms may contribute to increased creativity in patients with narcolepsy (D'anselmo et al., 2020; Lacaux et al., 2019).

### 2.4 Disturbed nighttime sleep

After EDS and cataplexy, DNS is the third most disabling symptom of narcolepsy and is more severe in NT1 than in NT2 (Barateau et al., 2024). Patients report multiple brief awakenings and generally poor sleep quality (Maski et al., 2022). Polysomnography (PSG) studies showed a correlation between DNS complaints and objective measures of sleep fragmentation (Barateau et al., 2022). Different measures were employed to quantify sleep fragmentation, so the results are not completely comparable (Maski et al., 2022). However, patients with NT1, compared with healthy controls, NT2 and other central disorders of hypersomnolence, tend to show reduced sleep efficiency, more non‐REM (NREM) stage 1 (while REM sleep duration mostly remains stable), more arousals, more transitions between wake and sleep both in REM and NREM stages, shorter sleep bouts, abnormal pattern of transitions between sleep stages (Barateau, Lopez, Chenini, Pesenti et al., 2020; Barateau, Lopez, Chenini, Rassu et al., 2020; Ferri et al., 2016; Maski et al., 2020; Maski, Colclasure et al., 2021; Pizza et al., 2015; Sorensen et al., 2013), and altered NREM sleep microstructure also reflected via reduced periods of the cyclic alternating pattern (Ferri et al., 2005; Terzano et al., 2006). An abnormal overlap between features of different sleep stages has also been described (Cesari et al., 2023; Stephansen et al., 2018; Vilela et al., 2024). These findings suggest that sleep fragmentation in NT1 consists of an instability of nocturnal sleep in its entirety, more than an impairment restricted to REM sleep.

### 2.5 Associated sleep disorders

Beyond the core clinical features, narcolepsy (mostly NT1) can present with other sleep disorders related to REM sleep or not.

The REM sleep parasomnias are frequent. Since Schenck and Mahowald recorded episodes of REM sleep behaviour disorder (RBD) in patients with narcolepsy (Schenck & Mahowald, 1992), the disease emerged as the second cause of RBD behind synucleinopathies and the most frequent form in the young population (Dauvilliers et al., 2018) and without biomarkers of neurodegeneration, in contrast with isolated RBD (Antelmi et al., 2019; Barateau et al., 2018). RBD is reported in up to 60% of patients with NT1, but REM sleep without atonia (RSWA) is also common with both increased tonic and phasic activity even in patients without clinical RBD symptoms (Dauvilliers, Rompré et al., 2007; Ferri et al., 2008). RBD and RSWA are common among paediatric patients with NT1 (Antelmi et al., 2017). In patients with NT1 and RBD, compared with patients with isolated RBD, motor episodes are more frequent, mild and stereotyped rather than violent, and distributed across all REM phases throughout the night and occasionally also during daytime naps (Antelmi et al., 2017, 2019; Cipolli et al., 2011; Ferri et al., 2021). In children, severe RBD correlates with more severe cataplexy, suggesting an impairment of REM‐related motor control that proportionally affects wakefulness and sleep (Antelmi et al., 2017). Often overlooked in clinical practice, patients with narcolepsy often complain of nightmares associated with more depressive symptoms (Leu‐Semenescu et al., 2022; Pisko et al., 2014) and are lucid dreamers more than healthy controls (Dodet et al., 2015). Also NREM parasomnias can frequently appear in patients with narcolepsy, with sleep‐related eating disorders being the most common (Leu‐Semenescu et al., 2022).

Restless legs syndrome (RLS) was reported in 15% of cases of narcolepsy with cataplexy and, in one‐third of these, symptoms of RLS also appeared during the daytime, while at bedtime they do not usually overcome sleepiness to impair sleep onset (Plazzi et al., 2010). Periodic limb movements during sleep (PLMS) are more frequent than in the general population. Particularly, the PLMS index is markedly increased during REM sleep (Dauvilliers, Pennestric et al., 2007) and, in comparison with idiopathic RLS, limb movements in narcolepsy present a reduced periodicity and are distributed across the entire night rather than prevail in the first part (Ferri et al., 2006), a finding evident also in NT1 occurring during childhood–adolescence (Ferri et al., 2018). In contrast with evidence on primary RLS, RLS in narcolepsy does not appear to be associated with reduced ferritin in serum or CSF, pointing to a different pathogenic mechanism (Barateau, Chenini, Lotierzo et al., 2020).

In the context of narcolepsy, also obstructive sleep apneas (OSA) can appear, possibly influenced by comorbid overweight and obesity (Jennum et al., 2013; Sansa et al., 2010), but, albeit frequent, apparently not affecting the sleepiness severity in NT1 (Pizza, Tartarotti, et al., 2013).

These overlapped sleep disorders could contribute to delaying the diagnosis and to the worsened nighttime sleep quality. However, a recent investigation did not disclose any association of RBD, RSWA, RLS, PLMS and OSA (taken independently) on perceived DNS, which is most probably of multifactorial origin (Barateau et al., 2022).

### 2.6 Other comorbidities

In NT1, disease burden is not limited to sleep‐related symptoms, and various comorbidities can present at disease onset, at the time of the diagnosis, and along the clinical course. Studies demonstrated associations with multiple endocrine, nutritional and metabolic comorbidities (dyslipidaemia, obesity, diabetes), hypertension, and increased risk of hypertension and major cardiovascular events (Barateau & Dauvilliers, 2023; Ben‐Joseph et al., 2023; Black et al., 2017; Jennum et al., 2013, 2017; Ohayon, 2013; Poli et al., 2009). Cardiovascular morbidity could be influenced by the normotensive non‐dipper profile of blood pressure and increased 24‐hr heart rate, demonstrated in untreated patients with NT1 (Dauvilliers et al., 2012; Grimaldi et al., 2012) and confirmed in paediatric NT1, pointing to an intrinsic feature of the disease since its onset (Vandi, Rodolfi et al., 2019). The blunted nocturnal pressure dipping has been associated with REM sleep duration and with the presence of SOREMPs (Dauvilliers et al., 2012). Psychostimulant medications for EDS also contribute to increased blood pressure (Bosco et al., 2018).

Psychiatric morbidity is also relevant, with an increased prevalence of depression and anxiety in comparison with the general population (Barateau, Lopez, Chenini, Pesenti et al., 2020; Dauvilliers et al., 2009; Ohayon, 2013). In NT1, anxiety and depressive symptoms appear associated with subjective DNS (Barateau et al., 2022). Narcolepsy and its possible comorbidities lead to impaired quality of life (Szakács et al., 2019; Vignatelli et al., 2011).

Paediatric narcolepsy can present a specific profile of comorbidity, with rapid weight gain and precocious puberty, typically at disease onset (Jennum et al., 2017; Plazzi et al., 2006, 2018; Poli et al., 2013; Ponziani et al., 2016), and psychological and cognitive disturbances often falling under the spectrum of attention‐deficit/hyperactivity disorder (Inocente et al., 2014a, 2014b; Lecendreux et al., 2015; Rocca et al., 2016; Simoncini Malucelli et al., 2024).

The determinants of this multifaceted profile of comorbidity are mostly unknown, but the dysfunction of orexin transmission could play a role (Mahoney, 2019).

## 3 REM SLEEP IN THE DIAGNOSIS OF NARCOLEPSY

### 3.1 Diagnostic criteria

Currently, NT1 and NT2 are diagnosed according to the 2023 ICSD‐3‐TR criteria reported in Table 1 (American Academy of Sleep Medicine, 2023), with a central role of SOREMP as a diagnostic marker since the first ICSD in 1990 (Diagnostic Classification Steering Committee, 1990). In the absence of cataplexy and orexin deficiency, an MSLT with reduced mean sleep latency and less than 2 SOREMPs is compatible with IH, which can be diagnosed also by total sleep time ≥ 660 min on a 24‐h PSG or actigraphy (American Academy of Sleep Medicine, 2014).

Patients with documented orexin deficiency present lower REM sleep latency and more SOREMPs during both nighttime and daytime, and shorter sleep latency during MSLT (Andlauer et al., 2012; Um et al., 2022). Nevertheless, the neurophysiological criteria of NT1 and NT2 are very similar, and they were identical in ICSD‐3 (American Academy of Sleep Medicine, 2014). Similarly, the definition of SOREMP (both nocturnal and diurnal) is the same for both diseases, that is, the appearance of one epoch of REM sleep within 15 min from the sleep onset as defined by the MSLT procedure (American Academy of Sleep Medicine, 2023).

Instead, the clinical significance of one isolated SOREMP during MSLT is still unclear. Formally it is considered a normal finding, especially if recorded during the first nap of MSLT, although a few data suggest that patients with one SOREMP have PSG and MSLT features more like patients with narcolepsy than with IH (Bozluolcay et al., 2015).

### 3.2 MSLT and REM sleep

The MSLT was identified as a useful diagnostic exam for narcolepsy at the end of the 1970s when it was employed to record reduced sleep latency and the presence of SOREMPs (Mitler et al., 1979; Richardson et al., 1978), and was subsequently standardized to improve its reliability (Krahn et al., 2021; Littner et al., 2005).

Despite being widely recognized as the neurophysiological gold‐standard for diagnosing pathological sleepiness, there are notable issues in the application of MSLT criteria.

The MSLT features of narcolepsy change as a function of age, with progressive lengthening of sleep latency and decreased number of SOREMPs (Dauvilliers et al., 2004; Lividini et al., 2021).

The MSLT was therefore validated in a paediatric population (in a bi‐centric study from two European sleep centres), and the current general criteria were confirmed with high accuracy for CSF orexin deficiency in children. In children, either a mean sleep latency ≤ 8.2 min or at least 2 SOREMPs showed an accuracy similar to the classic MSLT criteria (Pizza et al., 2019).

Another relevant issue in applying current MSLT criteria is the high test–retest variability over time. Both mean sleep latency and the number of SOREMPs can significantly change over time (Huang et al., 2018; Lopez et al., 2017; Ruoff et al., 2018; Sansa et al., 2014; Trotti et al., 2013). The poor consistency between exams performed at variable years' distance can lead to a modification of the diagnosis, which is far more common in patients without cataplexy (Lopez et al., 2017). This variability suggests the potential usefulness of repeating MSLT over time, and questions the diagnostic boundaries between NT2 and IH (Huang et al., 2018).

A recent study with machine learning cluster analysis of more than 1000 European patients with central disorders of hypersomnolence further challenged the concept of SOREMP as the distinctive feature of narcolepsy. In this study, patients without cataplexy were distributed between two clusters, where diagnoses of NT2 and IH were both widely represented. These two clusters were distinguished by the prevalence of sleep drunkenness, sleep inertia, unrefreshing naps, hypnagogic hallucinations and sleep paralysis, without differences in terms of MSLT mean sleep latency and number of SOREMPs, suggesting that the two phenotypes described by these clusters go beyond the conventional criteria of narcolepsy and IH (Gool et al., 2022).

Circadian rhythm disorders, shift workers and behaviourally induced insufficient sleep syndrome can mimic narcolepsy on the MSLT (reduced sleep latency and multiple SOREMPs; Goldbart et al., 2014). Therefore, PSG is required to rule out sleep deprivation the night before MSLT, and actigraphic monitoring before the execution of the exam can be further useful (Baumann et al., 2014). Other conditions, such as myotonic dystrophy type 1, can show MSLT with reduced sleep latency and SOREMPs, without evidence of relation to orexin system impairment (Dauvilliers & Laberge, 2012).

In SOREMPs of narcolepsy the sequence of progression to REM sleep is often characterized by direct transition from wakefulness to REM sleep or from N1 to REM sleep, whereas in mimics SOREMP typically appears from NREM stage 2 (N2; Drakatos, Kosky, et al., 2013; Drakatos, Suri, et al., 2013; Kawai et al., 2020; Liu et al., 2015). This altered transition pattern appears more frequently in NT1 than in NT2 (Drakatos, Kosky, et al., 2013), and was associated with HLA‐DQB1*0602 positivity (Kawai et al., 2020; Liu et al., 2015).

**FIGURE 1:**
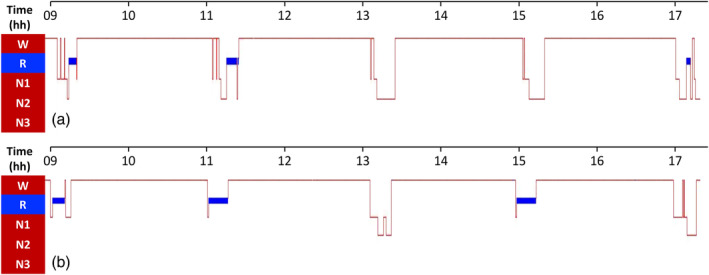
Multiple Sleep Latency Test (MSLT) in: (a) a patient with normal orexin (narcolepsy type 2 [NT2]) showing three sleep‐onset rapid eye movement (REM) periods (SOREMPs) from non‐REM (NREM) stage 2; and (b) in a patient with orexin deficiency (narcolepsy type 1 [NT1]) showing three SOREMPs from NREM stage 1, with short REM sleep latency and long REM sleep duration.

Alternative MSLT features have been explored. In the study by Pizza et al., a shorter latency to sustained sleep during MSLT was a sensitive and specific feature to identify narcolepsy with and without cataplexy against IH, which more often showed fluctuation between sleep and wake (Pizza et al., 2011). Murer et al. proposed REM sleep latency < 5 min and REM sleep duration > 40% during MSLT naps as more specific markers for narcolepsy (Murer et al., 2017). In a multicentric study from the European Narcolepsy Network, a machine learning approach identified the reduced REM sleep latency as promising predictor to correctly distinguish NT1 from NT2 (Zhang et al., 2018). Lopez et al. analysed multiple parameters from MSLT (including sleep latency, REM latency and duration, sleep duration, and transition pattern to REM sleep). They found that orexin deficiency was best identified among all patients with suspect central disorder of hypersomnolence by a mean REM sleep duration > 4.1 min, while at least one direct transition from wakefulness to REM sleep presented the highest sensitivity. Among all patients with narcolepsy, a REM sleep duration > 5.7 min identified orexin deficiency with lower yet satisfactory accuracy, due to a consistent proportion of patients with NT2 with long REM duration, (Lopez et al., 2023), again pointing to NT2 as an entity with variable phenotype either overlapped with NT1 or IH (Fronczek et al., 2020; Lammers et al., 2020). These results suggest the possibility to further refine the MSLT criteria to improve its accuracy for narcolepsy and to differentiate NT1 from NT2, as shown in Figure 1.

### 3.3 PSG and REM sleep

Polysomnography is an essential tool in narcolepsy that allows the identification of the nocturnal SOREMP, and the exclusion of confounding factors for NT2. Moreover, PSG can disclose REM and NREM‐related features of narcoleptic sleep: the presence of RSWA, RBD, NREM parasomnias, sleep fragmentation, PLMS, and comorbid sleep disorders such as OSA.

Since its discovery (Rechtschaffen et al., 1963; Vogel, 1960), the nocturnal SOREMP has been employed as a diagnostic feature of narcolepsy (Diagnostic Classification Steering Committee, 1990), removed from narcolepsy criteria in ICSD‐2, and then reintroduced in ICSD‐3 as complementary to MSLT results (American Academy of Sleep Medicine, 2005, 2014). Nocturnal SOREMP appears highly specific in predicting orexin deficiency, with a low sensitivity of 50%–60%, in adult and paediatric cohorts (Andlauer et al., 2013; Reiter et al., 2015). Thus, in 2023 ICSD‐3‐TR, nocturnal SOREMP, if associated with EDS and clear‐cut cataplexy, became a sufficient diagnostic marker of NT1, independent from the MSLT result (American Academy of Sleep Medicine, 2023). This change, indirectly, highlights the importance of correctly recognizing true cataplexy based on degrees of certainty (Table 2).

Other PSG features have been employed as supportive diagnostic markers in narcolepsy.

The combined presence of nocturnal SOREMP with the high percentage of nighttime spent in N1 and the high wakefulness–sleep transition index showed good accuracy for NT1, similar to that of MSLT criteria (Pizza et al., 2015). Going further, the analysis of nocturnal stage transitions, indices of more fragmented nocturnal sleep, showed good specificity for narcolepsy regardless of the presence of SOREMP (Christensen et al., 2015; Maski et al., 2020; Olsen et al., 2017). Quantitative analysis of PSG signals, expressed as hypnodensity metrics describing the blurred boundaries between sleep stages, provided promising results for distinguishing central disorders of hypersomnolence (Cesari et al., 2023; Stephansen et al., 2018; Vilela et al., 2024).

Daytime continuous PSG monitoring has been tested as a supportive diagnostic tool. The presence of daytime SOREMP during spontaneous naps was found to accurately predict the occurrence of two or more SOREMPs on MSLT (Pizza, Moghadam, et al., 2013). In paediatric patients, daytime SOREMP resulted similarly accurate to MSLT criteria in identifying NT1 (Pizza et al., 2024).

**FIGURE 2:**
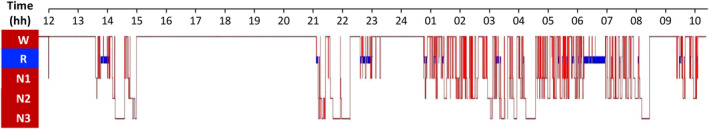
Daytime and nighttime continuous polysomnographic (PSG) recording of a patient with orexin deficiency showing daytime spontaneous naps with sleep‐onset rapid eye movement (REM) periods (SOREMPs), and nocturnal sleep with SOREMP and fragmented REM and non‐REM (NREM) sleep.

Additionally, RSWA yielded a good diagnostic performance for paediatric narcolepsy, with sensitivity being superior to specificity, variable on different cut‐offs employed (Bin‐Hasan et al., 2018). The combination of nocturnal REM latency with automatically computed RSWA provided an index with better diagnostic accuracy for paediatric NT1 than the two parameters alone (Silvani et al., 2021). In a small sample of adults with narcolepsy, RSWA showed moderate sensitivity and good specificity for identifying NT1 (Yon et al., 2021). A typical daytime and nighttime hypnogram of a patient with orexin deficiency is portrayed in Figure 2.

## 4 OREXIN DEFICIENCY AND ITS EFFECT ON REM SLEEP DYSREGULATION IN NT1

### 4.1 Orexin deficiency is the cause of NT1

Since the discovery of narcolepsy, the cause of the disease has been the subject of debate, with theories alternatively proposing an organic mechanism or a psychiatric disease (Droogleever Fortuyn et al., 2011). Also, Vogel interpreted the recording of early REM sleep onset from a psychoanalytical perspective (Vogel, 1960). In the 1980s, the discovery of the association of the disease with certain HLA class II alleles suggested an immune‐mediated mechanism (Billiard et al., 1986; Juji et al., 1984; Langdon et al., 1984).

In 2000, orexin deficiency was disclosed as the core cause of narcolepsy with cataplexy in humans, documented both in vivo in the CSF and post‐mortem in neuropathological studies (Nishino et al., 2000; Peyron et al., 2000; Thannickal et al., 2000). The causal role of orexin deficiency was supported by animal models (Lin et al., 1999; Ripley et al., 2001). There is not a perfect equivalence between orexin deficiency and narcolepsy with cataplexy: about 10% of patients with cataplexy show CSF orexin > 110 pg ml^−1^ (above the current definition of orexin deficiency, with radioimmunoassay according to the standard reference of the Stanford values), and cataplexy is absent in 10%–30% of narcolepsy with orexin deficiency (Baumann et al., 2014; Mignot et al., 2002). The meaning of CSF orexin value between 110 and 200 pg ml^−1^ (considered “intermediate”) is unclear, given that about half of these patients may present cataplexy, with more hallucinations and more SOREMPs, especially in the group below 150 pg ml^−1^ (Postiglione et al., 2022; Van Der Hoeven et al., 2022). The causes of narcolepsy in patients without CSF orexin deficiency are less defined, with a partial deficit of orexin transmission being hypothesized in NT2 but still debated (Thannickal et al., 2009).

The CSF orexin levels are typically normal in other central disorders of hypersomnolence and other sleep disorders (Dauvilliers, Baumann et al., 2003). Additionally, rare secondary forms of narcolepsy, both with and without cataplexy, exist, for instance in demyelinating diseases, in paraneoplastic encephalitis, or associated with genetic syndromes (e.g. Niemann‐Pick type C, mutations of DNA‐methyltransferase type‐I), with CSF orexin levels that can be normal, reduced or intermediate (Dauvilliers, Bauer et al., 2013; Moghadam et al., 2014; Nishino & Kanbayashi, 2005).

Although the disease mechanism of orexin deficiency is not completely understood, the current evidence strongly supports the hypothesis that, in NT1, there is acquired destruction of orexin‐producing neurons, localized in the posterolateral hypothalamus. The loss of orexinergic neurons is caused by autoimmune damage, possibly triggered by environmental factors like vaccines or infections and mediated by T‐cell activation (Latorre et al., 2018; Liblau et al., 2023). A genetic predisposition exists, mainly represented by the presence of the HLA‐DQB1*0602 allele (Kornum et al., 2017; Liblau et al., 2023; Ollila et al., 2023; Partinen et al., 2014). Other risk loci involved in the immune response have been associated with NT1, among which, polymorphisms of T‐cell receptor alpha and beta represent exceptions to other autoimmune diseases (Ollila et al., 2023).

### 4.2 Orexin deficiency in the regulation of REM sleep

In the currently accepted physiological theory, hypothalamic orexinergic neurons provide diffuse excitatory efferences involved in promoting and stabilizing arousal, suppressing REM sleep, and inhibiting REM sleep‐related muscle atonia during wakefulness (Mahoney, 2019).

In narcolepsy, according to animal models, orexin deficiency impairs the wake‐promoting activity of basal forebrain, locus coeruleus and tuberomammillary nucleus, favouring rapid transitions to NREM sleep and altering the stability of sleep–wake dynamics (Diniz Behn et al., 2010; Hasegawa et al., 2014; Mochizuki et al., 2004). Abnormal propensity to REM sleep during the daytime and in the first part of the night seems to depend on a disinhibition of REM sleep and a loss of circadian control, rather than an increased REM sleep pressure (Roman et al., 2018; Tafti et al., 1992). Recent evidence on murine models suggests that, physiologically, a subpopulation of orexinergic neurons is active on the pontine sublaterodorsal nucleus during REM sleep, contributing to its stability (Feng et al., 2020). Silencing of these REM sleep‐active orexinergic neurons provokes abnormal REM sleep architecture in mice, which could explain narcolepsy REM‐related symptoms (Ito et al., 2023).

Indirect signs of this dysregulation have been observed also in human NT1, with blurred boundaries between behavioural states, involving wake, REM and NREM sleep (Schoch et al., 2017; Stephansen et al., 2018), increased eye movements during REM sleep (Christensen et al., 2017), and by EEG spectral analysis showing higher frequency activity during SOREMP than during nocturnal REM sleep, as if SOREMP was dissociated from physiological sleep (Pizza et al., 2017). Lower levels of CSF orexin appear also to be associated with sleep fragmentation (Barateau, Lopez, Chenini, Rassu et al., 2020; Hansen et al., 2017), and with the presence of RBD and RSWA (Knudsen et al., 2010).

### 4.3 Orexin deficiency and cataplexy

The neural pathways explaining cataplexy resemble an aberrant activation of REM sleep muscle atonia during wakefulness, mediated by the sublaterodorsal nucleus, which is normally inhibited during wakefulness and NREM sleep by the ventrolateral periaqueductal grey area and lateral pontine tegmentum (Luppi et al., 2012). These latter nuclei receive excitatory inputs from the hypothalamic orexinergic neurons and γ‐aminobutyric acid (GABA)ergic inhibitory signals from the central nucleus of the amygdala. Sudden emotional stimuli activate neurons in the medial prefrontal cortex, subsequently stimulating hypothalamic orexinergic neurons and the central nucleus of the amygdala. In the absence of orexin, the unbalanced activation of the central nucleus of the amygdala could lead to abnormal disinhibition of the sublaterodorsal nucleus, resulting in transient muscle atonia during wakefulness (Burgess et al., 2013; Hasegawa et al., 2017; Mahoney et al., 2017; Oishi et al., 2013). Functional magnetic resonance imaging during cataplectic attacks supports the existence of this mechanism in human narcolepsy (Meletti et al., 2015; Vaudano et al., 2019). However, a partially different regulation of cataplexy and REM sleep has been hypothesized involving the serotoninergic system, alongside the orexinergic one. In fact, mice double knockout for orexin and pre‐synaptic serotonin transporter showed suppression of cataplexy but an increase in REM sleep (Seifinejad et al., 2020).

### 4.4 Orexin deficiency and other functions

Additionally, orexin is involved in reward behaviours, feeding and metabolism, and autonomic regulation by promoting sympathetic tone, mainly through the binding to the orexin‐receptor‐1 (Blouin et al., 2013). Disruption of these pathways in orexin‐deficient brains could explain, respectively, the increased depression, weight gain and metabolic syndrome, and blood pressure and cardiovascular dysfunction of narcolepsy (Mahoney, 2019). However, these mechanisms in narcolepsy have not been fully elucidated.

Moreover, REM sleep‐related phenomena cannot be explained entirely by orexinergic transmission deficiency, as they are present also in NT2, where no orexin loss is commonly detected.

## 5 TREATMENTS FOR NARCOLEPSY AND THEIR EFFECT ON REM SLEEP

Currently, only symptomatic therapies are available for narcolepsy. Besides non‐pharmacological approaches that include sleep hygiene and planned napping, pharmacological treatments can be distinguished in wake‐promoting agents, oxybates, and antidepressants (Bassetti et al., 2021; Maski, Trotti et al., 2021).

Alerting agents enhance central monoaminergic transmission through different mechanisms. Modafinil and its D‐enantiomer armodafinil enhance dopaminergic transmission, while solriamfetol, methylphenidate and amphetamine derivates variably enhance dopaminergic and noradrenergic transmission (Barateau & Dauvilliers, 2019; Zeitzer et al., 2006). Modafinil/armodafinil and solriamfetol are in the first line of therapy for EDS in adult narcolepsy. Conversely, amphetamine derivates and methylphenidate, which were the first drugs to show effectiveness on sleepiness in narcolepsy (Parkes & Fenton, 1973; Yoss & Daly, 1959), are considered second‐line choices in the latest European guidelines, due to potential major side‐effects and lack of evidence with randomized controlled trials (Bassetti et al., 2021).

Dopaminergic‐noradrenergic stimulants do not often improve cataplexy and other REM‐related symptoms such as hallucinations and paralysis (Bassetti et al., 2021), despite being effective on EDS in both patients with and without cataplexy (Dauvilliers, Shapiro et al., 2020). Sleep structure is not modified by stimulants. However, amphetamines at high doses were reported to reduce REM sleep duration and extend REM sleep latency (Mitler et al., 1993).

Pitolisant is a selective presynaptic histamine H3‐receptor inverse agonist that mainly activates CNS histaminergic neurons. It was demonstrated to improve EDS, cataplexy and hallucinations in patients with NT1 (Dauvilliers, Bassetti, et al., 2013; Szakacs et al., 2017). It is in the first line for EDS and cataplexy in adult and paediatric patients with narcolepsy (Bassetti et al., 2021), and it is approved in Europe for narcolepsy in patients of 6 years and older. In a study on a murine model of narcolepsy, pitolisant extended REM sleep latency reduced REM duration, and attenuated slow‐wave sleep. These effects were further enhanced by the combination with modafinil (Lin et al., 2008). The anti‐cataplectic effect of pitolisant is hypothesized to rely on an enhanced histaminergic transmission to the amygdala of the narcoleptic brain, from which GABAergic neurons project to pontine nuclei that regulate REM sleep atonia (Szakacs et al., 2017).

A beneficial effect on nocturnal sleep and daytime symptoms of narcolepsy with cataplexy was observed with the nightly administration of gamma hydroxybutyrate (GHB) since the 1970s (Broughton & Mamelak, 1979; Lammers et al., 1993; Mamelak et al., 1986). GHB acts as GABA receptor B agonist and may inhibit the activity of the noradrenergic neurons from the locus coeruleus during nocturnal sleep with a rebound effect of noradrenergic firing during daytime (Barateau & Dauvilliers, 2019). Sodium oxybate, the sodium salt of GHB administered twice nightly (Mamelak et al., 2004), is thus approved for the treatment of EDS and cataplexy in adults and children (older than 7 years) with NT1 (Bassetti et al., 2021; Maski, Trotti et al., 2021). The improvement of symptoms is dose‐dependent and persists with prolonged treatment (Lecendreux et al., 2022). Solid evidence shows that sodium oxybate increases slow‐wave sleep, reduces N1 and REM sleep duration, increases REM sleep latency, reduces arousals and awakenings (Boscolo‐Berto et al., 2012; Roth et al., 2024; Xu et al., 2019), consolidates nocturnal sleep and reduces stage shifts, including transitions from REM sleep to wakefulness or N1 sleep and vice‐versa (Roth et al., 2017). Similar effects on EDS, cataplexy, DNS and nocturnal sleep structure have been observed with new alternative formulations of oxybate, the lower‐sodium oxybate (Bogan et al., 2021; Dauvilliers, Bogan, et al., 2022) and the extended‐release formulation with once‐nightly administration (Dauvilliers, Mignot et al., 2023; Dauvilliers, Roth et al., 2023; Roth et al., 2022). Additionally, sodium oxybate improves motor dyscontrol during REM sleep, with a dose‐dependent reduction of muscle activity (Mayer et al., 2017) and, in children with NT1, it was demonstrated to reduce RSWA and RBD episodes (Antelmi et al., 2021).

Tricyclic antidepressants such as clomipramine and imipramine were the first drugs to be proven effective on cataplexy (Guilleminault et al., 1974), probably for pro‐noradrenergic effect (Zeitzer et al., 2006). Currently, serotonin‐noradrenaline reuptake inhibitors (e.g. venlafaxine, duloxetine) are preferred for the off‐label treatment of cataplexy due to the best tolerability and large efficacy with tricyclic antidepressant as the second choice (Aran et al., 2010; Bassetti et al., 2021; Jin et al., 2019). Antidepressants can also provide benefits for sleep‐related hallucinations and paralysis (Bassetti et al., 2021; Møller & Østergaard, 2009). Abrupt suspension of antidepressants can provoke status cataplecticus (Wang & Greenberg, 2013). In the open‐label phase of a trial of lower‐sodium oxybate, the gradual tapering and discontinuation of other anti‐cataplectic medications (mostly antidepressant) during the oxybate titration was accompanied by increased frequency of cataplexy (Dauvilliers, Šonka, et al., 2022). The use of antidepressant favours the appearance of RBD, RSWA and PLMS in narcolepsy, like in the general population (Busková et al., 2015; DelRosso et al., 2023; Ferri et al., 2023; Lee et al., 2016). Moreover, antidepressants may change REM sleep latency (Wichniak et al., 2017), possibly affecting the number of SOREMPs and altering the diagnostic PSG and MSLT for narcolepsy. REM‐suppressing antidepressants (mainly selective serotonin reuptake inhibitors, serotonin‐norepinephrine reuptake inhibitors and tricyclics) are more likely to give false negative results, especially if taken in the morning, while NREM‐suppressing antidepressants (i.e. bupropion, mirtazapine) can increase SOREMPs leading to false positive MSLT (Mignot et al., 2006).

As orexin deficiency is the core pathological mechanism of NT1, intrathecal administration of orexin was tested in murine models. Continuous orexin infusion provided improvement in cataplexy and reduction of SOREMPs in orexin‐knockout mice, confirming the hypothesis of persistent functioning of orexin‐receptors in narcolepsy (Kaushik et al., 2018). The blood–brain barrier is impermeable to orexin, thus, in recent years, small‐molecules orexin‐receptor‐2 agonists have been developed (Irukayama‐Tomobe et al., 2017; Sun et al., 2023; Yukitake et al., 2019). The intravenous administration of orexin‐receptor‐2 agonists increased and stabilized wakefulness, decreased NREM and REM sleep, cataplexy episodes, and abolished SOREMPs in murine models, improved EDS in humans with NT1 and NT2, and reduced cataplexy in humans with NT1 (Evans et al., 2022). The first phase II trial of an oral orexin‐receptor‐2 agonist in patients with NT1 showed great improvements in EDS and cataplexy, but the trial was early terminated due to hepatotoxic adverse events (Dauvilliers, Mignot et al., 2023). Other clinical trials with novel alternative molecules are currently ongoing. The orexin‐receptor‐2 agonists are, in perspective, potentially useful to improve a wide range of symptoms of narcolepsy, although it must be elucidated their impact on REM sleep and all non‐sleep‐related symptoms. It is possible that these molecules will not entirely solve the spectrum of NT1 symptoms, given the lack of activation of the orexin‐receptor‐1. The development of orexin‐receptor‐1/dual orexin‐receptor agonists could represent an option to re‐integrate all the orexinergic‐deficient pathways, although safety concerns regarding addiction and blood pressure exist (Saitoh & Sakurai, 2023).

## 6 CONCLUSION

Historically, narcolepsy has been regarded as a disease of altered REM sleep, and it is still unquestionable that the impairment of REM sleep regulation is crucial to understanding symptoms, pathological mechanisms, neurophysiological exams, and the effect of medications. However, advances in this research field challenged the equivalence between narcolepsy and REM sleep alteration. The dysfunction of REM sleep secondary to the orexin deficiency cannot explain important phenomena of the condition, such as sleep fragmentation with NREM sleep, sleep attacks without SOREMP, and metabolic and psychiatric comorbidities. Conversely, the mechanisms of REM‐related symptoms in NT2 are still unknown, possibly relying on a partial or transient dysfunction of the orexinergic system or the involvement of different pathways. Moreover, the association of SOREMPs with other typical narcoleptic features, in the absence of cataplexy and orexin deficiency, has been questioned, suggesting that, except for disease‐defining SOREMPs, NT2 is a heterogeneous entity, partly overlapped between NT1 and IH phenotypes. From another perspective, the study of REM sleep‐related phenomena in the models of orexin‐deficient NT1 contributed to the knowledge of physiological REM sleep mechanisms. A deeper understanding of NT1 and NT2 is thus needed, to explore further the extent of REM sleep dysregulation and the other mechanisms independent from it, and its potential reversibility with orexin‐receptor agonists.

## AUTHOR CONTRIBUTIONS

**Francesco Biscarini:** Conceptualization; writing – original draft. **Lucie Barateau:** Writing – review and editing. **Fabio Pizza:** Writing – review and editing. **Giuseppe Plazzi:** Writing – review and editing. **Yves Dauvilliers:** Writing – review and editing; conceptualization; supervision.

## CONFLICT OF INTEREST STATEMENT

F.B. received support for attending congresses by Bioprojet. L.B. received support for attending meetings by Bioprojet, Idorsia, and Linde, received consulting fees by Bioprojet, Takeda, Idorsia, and Jazz, received honoraria for lectures by Bioprojet, Takeda, and Idorsia, participated on boards for Bioprojey and Idorsia. F.P. received support for attending congresses by Bioporjet, received honoraria for lectures by Jazz and Bioprojet, participated on boards for Takeda. G.P. is member of Advisory board for Jazz, Bioprojet, Orexia, Takeda, Idorsia. Y.D received founds for attending meeting by Jazz, Bioprojet, and Avadel, received honoraria for lectures by Jazz, Bioprojet, Takeda, UCB, Orexia, Idorsia, and Avadel, participated on boards for Idorsia.

## Data Availability

Data sharing not applicable to this article as no datasets were generated or analysed during the current study.
